# Psychiatric Comorbidity in Neurological Disorders: Towards a Multidisciplinary Approach to Illness Management in the United Arab Emirates

**DOI:** 10.3389/fpsyt.2019.00263

**Published:** 2019-04-25

**Authors:** Taoufik Alsaadi, Seada Kassie, Ola Mohamed Ali, Khaldoun Mozahem, Safana al Fardan, Ahmed M. Ahmed

**Affiliations:** ^1^Department of Neurology, Psychiatry, American Center for Psychiatry and Neurology, Abu Dhabi, United Arab Emirates; ^2^Department of Education - Medical Affairs, Shaikh Khalifa Medical Center, Abu Dhabi, United Arab Emirates

**Keywords:** psychiatric comorbidity, neurological disorders, illness management, multidisciplinary approach, integrated collaborative medical services, United Arab Emirates

## Abstract

**Aim:** To determine the prevalence of mood and anxiety disorders in undiagnosed patients attending neurological services, and detect rates of referral to and attendance of psychiatric services.

**Methods:** Depressive symptoms and anxiety were assessed in 395 adult patients with primary diagnoses of neurological disorders. The Patient Health Questionnaire nine-item depression scale (PHQ-9), and Generalized Anxiety Disorder seven-item scale (GAD-7) were administered. Demographic details of the patients were recorded. Referral to and attendance of psychiatric services were recorded for patients scoring within the clinical range of depression and anxiety disorders (scores > 10).

**Results:** There was a 39% prevalence rate of depressive symptoms, 34% rate of anxiety, and 35.4% concurrent rate of both disorders in this cohort. The referral rate to psychiatric services was 33.6%, and attendance rate was 47.8%. There was significant association between severity of psychiatric symptoms and referral to psychiatric services, as well as significant association between comorbid psychiatric symptoms and attendance to psychiatric services.

**Conclusion:** Our results indicate similar prevalence rates of comorbid psychiatric symptoms to studies carried out in the Middle East and North Africa (MENA) region and relatively high attendance and referral rates to psychiatric services.

**Implications:** The results shed light on the clinical profile of patients in this region and support the need for integrated collaborative medical services. Moreover, findings have important implications for health care policies pertaining to resource distribution and funding.

## Introduction

Medical care has traditionally been dominated by a separation between disciplines catering to physiological symptoms versus those catering to psychological symptoms (1). Emerging research points to psychiatric comorbidity in the primary care setting and in specialized services, especially with chronic conditions like cardiovascular disease and diabetes. Among psychiatric comorbidities, depression and anxiety disorders are the most prevalent and have been found to hinder treatment response, increasing disability and reducing functional outcome (2–6). Given the shared etiological processes between neurological disorders and psychiatric disorders, reports of 50% prevalence rates of depression and anxiety among neurology patients are not surprising (7). Despite ample evidence on comorbidity, the provision of psychiatric intervention for such patients remains lacking.

Several epidemiological studies have attempted to quantify psychiatric comorbidity in primary care and specialized services. In a Hungarian large-scale national community survey, Purebl et al. (8) reported that 52% of those with cardiovascular disease displayed symptoms of depression and anxiety, with 30% meeting the diagnostic criteria for depression. Similarly, a Belgian cross-sectional survey of primary care practices in the country revealed that although 5.4% of the 2,316 patients surveyed reported psychiatric complaints, 42.5% had an unreported psychiatric comorbidity (2). Mood and anxiety disorders were the most prevalent. Complications from diabetes are most prevalent in the presence of psychiatric disorders (6), and mortality rates increase in individuals with myocardial infarction if they also suffer from anxiety (7). Among 300 neurology patients surveyed in the UK, 47% met the criteria for a *Diagnostic and Statistical Manual of Disorders, Fourth Edition* (DSM-IV) diagnosis of depression and anxiety (4). In a Canadian community sample, individuals diagnosed with epilepsy were more likely to experience anxiety symptoms and had higher rates of suicidal ideation (9), and those with migraine were more likely to experience major depressive disorder, bipolar disorder, panic disorder, and social phobia (10). Moreover, it is often found that the patients with psychiatric symptoms are also those with the most disability and the least response to treatment, and are the most difficult to manage. They are also often the most frequent attendants in primary care services (5).

A multidisciplinary approach to disease management and education in primary care has been instrumental in managing the epidemic of psychiatric comorbidity in physical illnesses (11). While efforts are being seen in other parts of the world highlighting the need, such data is lacking in the United Arab Emirates (UAE). In recent years, studies have been carried out in the Arab Gulf region looking at prevalence and correlates of psychiatric disorders in patients attending primary care. Among those, one study found that 30% of patients with epilepsy showed clinically significant symptoms of depression and anxiety (12). Similarly, a study looking at psychiatric comorbidity in patients with type 2 diabetes in the UAE found a 12.5% prevalence rate of mental health concerns and a significant correlation between patients’ mental health status and their primary diagnosis (13). Two independent studies looking at the impact of depression and anxiety on health-related quality of life among patients with a) epilepsy and b) multiple sclerosis found that depression and anxiety, along with the use of antidepressants, were among the highest predictors of patients’ health-related quality of life (14, 15). A study that looked at the prevalence of psychiatric comorbidity in 1,046 primary health clinic attendees in Kuwait found a comorbidity rate of 20.4% between depressive symptoms, anxiety, and somatization (16); 42.7% of patients suffered from at least one type of disorder, while 11% had two, and 10.4% had symptoms of all three psychiatric disorders. Al-Otaibi and colleagues (17) found a 37.1% rate of depression in patients attending primary care in Kuwait, while Al-Qadhi et al. (18) found a 49.9% rate of depression in a similar cohort in Saudi Arabia.

While such studies are a promising start for the wider Gulf region, high prevalence rates of psychiatric disorders indicate the need for a multidisciplinary approach to disease management and provision of integrated health services. According to the Mental Health Atlas (19), there is inadequate provision and high stigmatization of mental health services in the Middle East. In the UAE, limited research in this area accounts for why medical practice remains behind in the implementation of a multidisciplinary approach. As such, the current study aims to bridge this gap by investigating the prevalence of concurrent depression and anxiety symptoms among patients attending only neurology services at a local outpatient center offering both psychiatric and neurological services. The current study capitalizes on the availability of these conjoint services to determine the prevalence of mood and anxiety disorders in patients seeking only neurological services at the center. Moreover, the study looks at whether concurrent depressive and anxiety symptoms are detected by the attending neurologist based on patient self-report, the severity threshold at which referral to psychiatric services does occur, and whether referred patients attend these services.

## Methods

**Figure 1:**
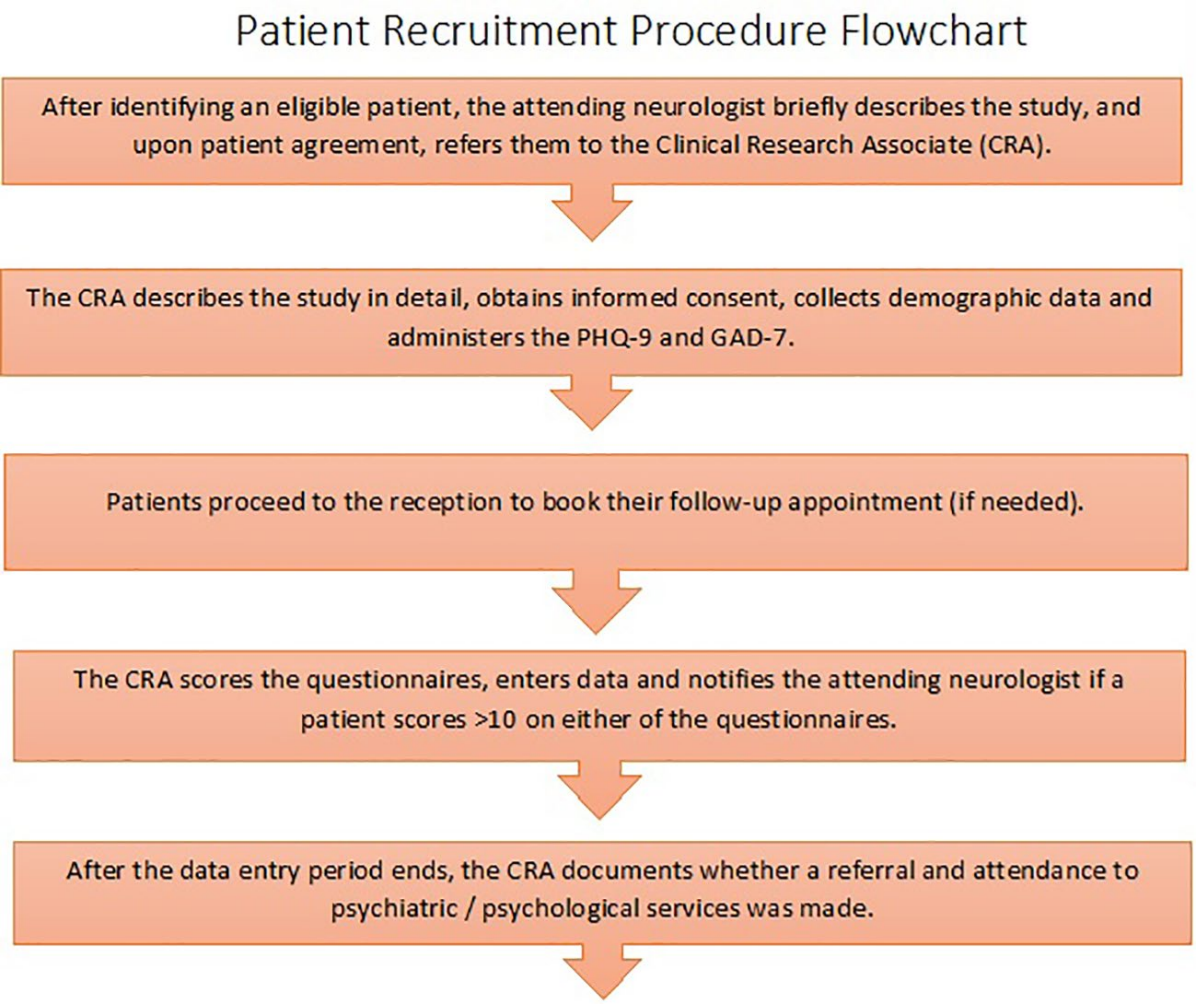
Patient recruitment procedure.

The study was granted ethics approval by the institutional review board at the American Center for Psychiatry and Neurology, Abu Dhabi, UAE. Patients were recruited between September 2016 and November 2017 and signed a written informed consent form to participate in the study. A chart displaying the patient recruitment procedure is shown in **Figure 1**.

### Patients

A total of 395 Arabic- and English-speaking patients between the ages of 18 and 65 with a current diagnosable neurological disorder were included in this study. Patients who had a diagnosis of, or were being treated for, one or more psychiatric disorders were excluded from the study. Between September 2016 and November 2017, a total of 424 patients were approached to participate in the study. Only 395 agreed to participate and were included in the study.

### Instruments

The Patient Health Questionnaire nine-item depression scale (PHQ-9) is a brief, self-administered tool for the screening and diagnosis of depression (20). It is based on the nine DSM-IV criteria for depression and can detect depression outcome and changes over time (21). The total score can range from zero to 27, and a score of >10 is indicative of the presence of depression. The PHQ-9 depression scale has been validated in the general population (22). Both the English and the validated Arabic (23) versions of the questionnaire were used in the study.

The Generalized Anxiety Disorder 7-item scale (GAD-7) (24) was used to screen for general anxiety disorder. It consists of a brief, seven-item questionnaire, and a score of >10 is indicative of the presence of generalized anxiety disorder. It has been validated for use in the general population (25). Both the English and the validated Arabic (26) versions were used in the study.

### Data Collection and Safety Considerations

Patients were invited to complete the questionnaires at the end of their visit to the neurology clinic. To ensure patient safety, the clinical research associate routinely notified the attending neurologist if a patient scored >10 on either of the questionnaires; the attending neurologist then offered the patient referral to psychiatric services. Referral to psychiatry/psychology services by the neurologist and patient attendance were then recorded.

### Statistical Analysis

Statistical Package for Social Sciences (SPSS), Version 22 (27), was used to analyze collected data. Descriptive statistics were used to determine a) the proportion of patients scoring in the clinically significant range, b) the proportion of those patients referred to psychiatric services, and c) the proportion of those referred who attended psychiatric services. Additionally, further analysis was done to determine the relationship between comorbid psychiatric symptoms and referral to psychiatric services, and again between comorbid psychiatric symptoms and attendance to psychiatry/psychology services.

## Results

A total of 395 (F: 55.2%, M: 44.8%) patients ranging between ages 18 and 64 (M = 36.14, SD = 9.71) visiting the neurology clinic at the American Center for Psychiatry and Neurology were enrolled in the study between September 2016 and November 2017. Sociodemographic characteristics of the sample are displayed in **Table 1**. Descriptive analysis was carried out for the following variables: sex, nationality, ethnicity, patient status, primary diagnosis, severity of psychiatric symptoms, referrals to psychiatric services, and psychiatric treatment. Emirati patients made up 36.7% of those enrolled, while non-Emiratis accounted for 63.3%. Of those enrolled, 319 (80.8%) were of Arab ethnicity, while the rest 76 (19.2%) were of various ethnic backgrounds, including African, Caucasian, South Asian, and Southeast Asian. There were a total of 219 (55.4%) new patients visiting the center for the first time, while the remaining 176 (44.6%) were returning patients. Of those enrolled, 102 (25.8%) had a primary diagnosis of headache (73.5% migraine with and without aura and 26.5% tension-type headache), 91 (23.1%) had epilepsy, 19 (4.8%) had multiple sclerosis, and the rest 184 (46.3%) had a variety of conditions classified under “other neurological disorders.” These included Parkinson’s disease, diabetes mellitus with neurological manifestations, unspecified musculoskeletal disorders, malignant neoplasm of the brain, traumatic spondylopathy, cervical disk displacement, type 2 diabetes mellitus with polyneuropathy, myalgia, transient alteration of awareness, nondiabetic proliferative retinopathy, essential tremor, syncope and collapse, spasmodic torticollis, Bell’s palsy, cerebrovascular disease, cerebral aneurysm, and benign paroxysmal vertigo.

**Table 1:** Sociodemographic characteristics of the sample (*n* = 395).

**Age (years)**	**Mean: 36.14 ± 9.71**
	Range: 18–64
**Sex**	F: 218 (55.2%)
	M: 177 (44.8%)
Employment status	
Employed	216 (54.7%)
Unemployed	73 (18.5%)
Student	24 (6.1%)
Other	82 (20.8%)
Marital status	
Single	129 (32.7%)
Married	253 (64.1%)
Divorced	12 (3.0%)
Widowed	1 (.3%)
Nationality	
Emirati	145 (36.7%)
Non-Emirati	250 (63.3%)
Ethnicity	
African	4 (1.0%)
Arab	319 (80.8%)
South Asian	13 (3.3%)
Southeast Asian	11 (2.8%)
Caucasian	47 (11.9%)
Other	1 (.3%)
Primary diagnosis	
Epilepsy	91 (23.0%)
Headache	102 (25.8%)
Multiple sclerosis	19 (4.8%)
Other neurological disorders	183 (46.3%)
Psychiatric comorbidity	
Yes	140 (35.4%)
No	255 (64.6%)

Of the total 395 patients, 140 (35.4%) scored positively for concurrent symptoms of depression and anxiety (i.e., PHQ-9 and GAD-7 scores of >10). Seen individually, 154 (39%) patients showed symptoms of clinical depression, while 137 (34%) patients showed symptoms of generalized anxiety disorder within the clinical range. Of the 140 patients with concurrent symptoms of both disorders, 47 (33.6%) were referred to psychiatric services available at the center. There were a total of 67 (47.8%) patients receiving psychiatric treatment at the time of data analysis, 41 (61.2%) with and 26 (38.8%) without a referral from their attending neurologists.

Pearson’s chi-square tests were carried out to investigate the relationship between comorbid psychiatric symptoms and referrals to psychiatric services, as well as the relationship between comorbid psychiatric symptoms and attendance to psychiatric services. The same was done for primary diagnoses and comorbid psychiatric symptoms, as well as referrals/attendance to psychiatric services. There was a very significant association between severity of psychiatric symptoms and referral to psychiatric services (*X* ^2^ (1) = 24.96, *p* < .001). Seen separately, there was also a significant association between depressive symptoms and referral to psychiatric services (*X* ^2^ (4) = 10.86, *p* < .05), as well as a very significant association between symptoms of generalized anxiety disorder and referral to psychiatric services (*X* ^2^ (4) = 18.77, *p* < .001). There was also a very significant association between comorbid psychiatric symptoms and attendance to psychiatric services (*X* ^2^ (1) = 46.21, *p* < .001).

There was no significant relationship between primary diagnosis of neurological disorders and comorbid psychiatric symptoms. Nevertheless, 31.6% of the patients with a primary diagnosis of multiple sclerosis showed comorbid psychiatric symptoms. The same observation was made for 30.8% of patients with a primary diagnosis of epilepsy, 37.3% of patients with headache, and 34.1% of patients with diagnoses of a variety of other neurological disorders. Of the 102 patients with a primary diagnosis of headache, 75 (73.5%) were categorized under migraine and 27 (26.5%) were categorized under tension-type headache. Thirty (40%) of the patients in the migraine category showed comorbid psychiatric symptoms, while seven (25.9%) of those in the tension-type category showed similar symptoms.

## Discussion

This study was the first step toward cross-sectional epidemiological research looking at psychiatric disorders in neurology outpatients in the UAE. It looked at prevalence rates of psychiatric comorbidity and referral rates in patients attending dedicated neurology clinics. The current study’s findings of a 35.4% prevalence rate are not much different from similar, albeit larger, epidemiological studies (16–18) in the Arab Gulf region that looked at the prevalence of psychiatric disorders in patients attending primary care services. They found rates ranging between 37.1% and 49.9%, but two of these studies only measured depression. The 30.8% prevalence among patients with epilepsy supports the findings of Alsaadi et al. (12), who found the same rate of psychiatric comorbidity in a different UAE cohort. It must be said that these findings are slightly lower than those of similarly designed studies, which had prevalence rates between 47% and 55.1% (4, 28). A study that looked at neurology inpatients found a 51.3% prevalence rate of psychiatric disorders (29), while another study (30) looking at neurology outpatients found that 30% of patients referred to a neurologist had symptoms not explained by organic disease.There is a possible explanation for the slightly lower prevalence rate and high referral/attendance rates of psychiatric disorders in the current cohort. Data was collected from a center that caters to patients with psychiatry and neurology disorders. It can be argued that in medical facilities where specialized services are not available, prevalence rates would be higher and referral/attendance rates much lower than what was found in a facility that is geared toward providing integrated mental health care services. In the absence of proper screening for and diagnosis of psychiatric disorders, patients presenting with psychiatric symptoms may report them using somatic terms, leading to poor prognosis, frequent attendance to primary care services, increased resource utilization, and increased health care costs (5). Alkhadhari et al. (16) reported somatization as the most common psychiatric illness among their cohorts in a primary care setting, possibly due to the patients’ tendency to report their symptoms using physical terms. They also argued that due to the cultural stigma attached to psychiatric illnesses in the region, patients prefer attending primary clinics rather than secondary or tertiary mental health care facilities. Findings from the current cohort support the need for a multidisciplinary approach to managing neurological disorders. It can be argued that access to a multi-/interdisciplinary clinical team will lead to the timely and accurate diagnosis of psychiatric illnesses, which may otherwise present as somatization. It can also lead to infrequent use of primary care services and the lowering of health care costs and resource utilization. Evidently, there is a link between patient cost-sharing patterns and usage of psychiatric services in the UAE, where 36% of the total costs of ambulatory neuropsychiatric services are paid directly by patients (31). Policies toward the regulation of health insurance plans can be adapted to include neuropsychiatric services in all basic and enhanced insurance plans.

Another important finding of this study is the 35.4% concurrence of depressive symptoms and anxiety. Studies have shown that comorbidity between psychiatric disorders has been found to cause greater disability levels when compared to patients with a single psychiatric diagnosis (32). In a review by Hirschfeld (33), patients with concurrent depression and anxiety disorders took longer to respond to treatment, had slower recovery, utilized more medical resources, and had higher rates of recurrence and psychological disability than patients presenting with either disorder alone. In this cohort, a 39% prevalence rate of depressive symptoms and 34% anxiety disorders was found, while 35.4% had concurrent occurrence of both disorders. Symptoms are found to overlap in many cases, and this necessitates careful discrimination between differences for a proper diagnosis and treatment plan. The current study highlights the need for proper and timely screening of psychiatric disorders.

Lastly, the current cohort had 25.8% of patients with a primary diagnosis of headache—the second largest group after patients classified under “other neurological disorders.” Migraine sufferers made up 73.5% of that group and showed higher rates of psychiatric comorbidity than those who suffered from tension-type headache. In the past, clinical studies that compared these two types of headache patients did not observe significant differences in psychiatric comorbidity (34–36). More clinical studies with a larger sample size are needed to corroborate these findings.

### Limitations

This study has only looked at two types of psychiatric disorders, which could have limited the chances of screening for other psychiatric illnesses such as somatization and posttraumatic disorder (37, 38). Moreover, confounding factors such as smoking and substance abuse that could have shed more light on the characteristics of this cohort, were not documented. Although the sample size is not insignificant, multicenter studies with a much larger sample size are needed in order to create an epidemiological database in the UAE.

## Conclusion

This study investigated the prevalence of depressive and anxiety symptoms across neurological complaints in patients of this specialized service in the UAE. The results indicate slightly lower rates of prevalence of psychiatric comorbidity with neurological disorders and high rates of attendance and referral rates to psychiatric services. Based on the findings, the 33.6% referral rate and 47% attendance rate are promising, but more needs to be done in delivering integrated care to patients and the timely screening for psychiatric illnesses. The results shed light on the clinical profile of patients in this region and support the need for integrated collaborative medical services. Moreover, findings have important implications for health care policies pertaining to resource distribution and funding.

## Ethics Statement

This study was carried out in accordance with the recommendations of the institutional review board at the American Center for Psychiatry and Neurology. The protocol was approved by the ACPN IRB.

## Author Contributions

All authors have made a substantial contribution to the design, data collection, and analysis of the research and the drafting of the manuscript and have reviewed and accepted the contents of the manuscript prior to its submission. TA contributed in recruiting patients for participation and reviewing the manuscript. SK collected, entered, and analyzed data and contributed in preparing the manuscript. OM contributed in designing the study, prepared the study protocol for IRB approval, and reviewed the manuscript. KM contributed in recruiting patients for participation and reviewing the manuscript. SA collected data. AA contributed in designing the study and reviewed the manuscript.

## Conflict of Interest Statement

The authors declare that the research was conducted in the absence of any commercial or financial relationships that could be construed as a potential conflict of interest.
